# Artificial Intelligence-Based Localization of Small Bowel Anatomic Transition Zones in Crohn’s Disease Using Capsule Endoscopy

**DOI:** 10.1055/a-2884-1351

**Published:** 2026-06-12

**Authors:** Raphaëlle Rouveyre, Tristan Gomez, Harold Mouchère, Arnaud Bourreille, Catherine Le Berre

**Affiliations:** 1 Institut des Maladies de l’Appareil Digestif (IMAD), Hépato-Gastro-Entérologie et Assistance Nutritionnelle, Inserm CIC 1413 26922 Nantes Université, CHU NantesNantesPays de la LoireFrance; 2 Ecole Centrale Nantes, CNRS, LS2N, UMR 6004 27045 Nantes Université, F-44000NantesPays de la LoireFrance

**Keywords:** inflammatory bowel disease, anatomic landmarks, segmentation, AI, CNN, computer-aided

## Abstract

**Background** Small bowel (SB) capsule endoscopy (SBCE) is a key tool for Crohn’s disease (CD), but interpretation is time-consuming. Artificial intelligence (AI) could assist, yet automated SB segmentation, a prerequisite for analysis, may be challenging in patients with CD.

**Aims** To evaluate an AI model detecting the pylorus and ileocolonic junction in CD SBCEs.

**Methods** SBCE videos from 60 patients with CD were annotated by three readers. A ResNet-18 convolutional neural network classified frames into stomach, SB, or colon, with Viterbi-based anatomically constrained segmentation, using five-fold cross-validation. Accuracy was assessed by discrepancies relative to expert annotations. Univariate analysis explored associated factors. Expert review was conducted for poorly localized cases.

**Results** The model was trained on 596,980 images, including 426,674 SB images. Median localization error was 2 frames (1 s) for the pylorus and 118.5 frames (~5 min) for the ileocolonic junction. In 26 videos (43.3%), the model localized both junctions with a temporal discrepancy <1 min compared with experts. In 53.3% of cases, AI detection of the ileocolonic junction preceded expert annotation by a median of 46 min, potentially leading to missed terminal ileal lesions. No factors were significantly associated with localization accuracy. Expert review highlighted inadequate cleanliness, capsule stagnation, and back-and-forth movements as main contributors to poor AI performance.

**Conclusions** Our model successfully localizes both SB junctions in nearly half of SBCE videos from patients with CD. It consistently identifies the pylorus but shows modest performance at the ileocolonic junction. Capsule dynamics and bowel cleanliness remain major challenges for automated SB segmentation.

## Introduction

Small bowel (SB) capsule endoscopy (SBCE) has become a key modality for the diagnosis and monitoring of Crohn’s disease (CD). Despite its proven value, SBCE remains underutilized in clinical practice, with gastroenterologists more frequently favoring magnetic resonance enterography for SB assessment in patients with CD. However, SBCE has consistently demonstrated a higher diagnostic yield than cross-sectional imaging techniques for the detection of mucosal lesions, especially in the jejunum, as it offers unparalleled visualization of the mucosal surface throughout the entire SB. 1

One major barrier to the broader implementation of SBCE in both the diagnostic and monitoring settings is the time-consuming nature of image interpretation. Each examination requires approximately 30–60 min of sustained attention, and reader fatigue may occur even after a single capsule study, potentially increasing the risk of missed lesions and interobserver variability. 2 3 Artificial intelligence (AI), particularly convolutional neural networks (CNNs), has emerged as a solution to streamline SBCE interpretation, with demonstrated benefits in lesion detection and characterization of inflammatory features. 4 5 6 These systems have consistently demonstrated high diagnostic accuracy, reducing reading time, standardizing disease activity scoring, and assisting clinical decision-making. 7 8 9 10 11

Commercial computer-aided detection tools with validated performance in multicenter studies are now available for routine clinical use, including the SmartScan (OMOM, Jinshan Science & Technology, Chongqing, China) and ProScan (NaviCam, AnX Robotica, Plano, Texas, United States). 12 13 The European Society of Gastrointestinal Endoscopy now states that “AI/machine-learning algorithms may be used as an adjunct to conventional capsule reading, when available.” 14

However, these tools still require manual annotation of the pylorus and the ileocolonic junction. Indeed, anatomic segmentation of the gastrointestinal tract into stomach, SB, and colon represents a critical methodologic step for AI-based systems. While this task is intuitively performed by experienced readers, automated tools rely on accurate segmentation as a prerequisite for lesion localization and downstream image processing. Errors at this stage may propagate through subsequent analytic steps, ultimately degrading the performance of automated detection algorithms.

Existing models have not been specifically trained in patients with CD. 15 In this population, terminal ileitis and delayed gastric emptying are common features, 16 17 both of which increase the risk of capsule stagnation and repetitive back-and-forth movements. This may make accurate identification of the anatomic landmarks of the SB particularly challenging for AI-based systems.

The present study aimed to evaluate the performance of a homemade CNN coupled with a Viterbi algorithm for the automated detection of both SB landmarks across entire SBCE recordings in a population of patients with CD.

## Methods

### Preliminary ResNet-18 Model Testing

In a preliminary analysis performed on 27 SBCE examinations from retrospectively selected patients with CD, we assessed three optimized AI-based ResNet-18 models designed to classify each SBCE frame into three anatomic categories (“Stomach,” “Small Bowel,” “Colon”). The dataset was randomly split into three subsets using a conventional 50/25/25 ratio. Data augmentation was applied to the training set by introducing random horizontal or vertical flips; the test set remained unaltered to ensure unbiased evaluation. The most effective configuration was a ResNet-18 architecture trained with a mean squared error loss function, a dropout rate of 0.1 (i.e., deactivating one neuron in ten), and a weight-decay factor of 10 ^−6^ , which provided the best performance on both the validation and test sets ( **Supplementary Table 1** ). This optimized ResNet-18 architecture was subsequently applied to anatomic landmark detection tasks.

### SBCE Dataset

All SBCE films included in the present study were derived from the CROHN-PREP randomized controlled trial conducted between June 2022 and April 2023, which aimed to compare the efficacy of a simplified preparation to a polyethylene glycol (PEG)-based preparation in patients with established CD. 18 All SBCE were performed using the PillCam SB3 system (Medtronic), each comprising approximately 30,000 images with a resolution of 256 x 256 pixels. As part of the CROHN-PREP trial, the SB transit time (SBTT) was collected, defined as the length of time between the first duodenal image and the first cecal (or colonic) image, and the quality of bowel cleanliness was evaluated using the validated KODA score. 19 20

### Ethics

Patient information was given orally and in writing, then oral informed consent was obtained from each patient and notified in the patient’s clinical record before the enrolment in the trial. The data collected in this study were processed electronically in accordance with the requirements of the Commission Nationale de l'Informatique et des Libertés (CNIL), with reference methodology MR001. The trial, together with this ancillary study, was approved by the “Comité de Protection des Personnes” (CPP) Nord Ouest IV research ethics committee on the January 21, 2022 under the reference 21.03307.000028, and was registered and posted on the ClinicalTrials.gov public website (NCT05117996). Videos were pseudonymized prior to analysis.

### Film Annotation

**Fig. 1:**
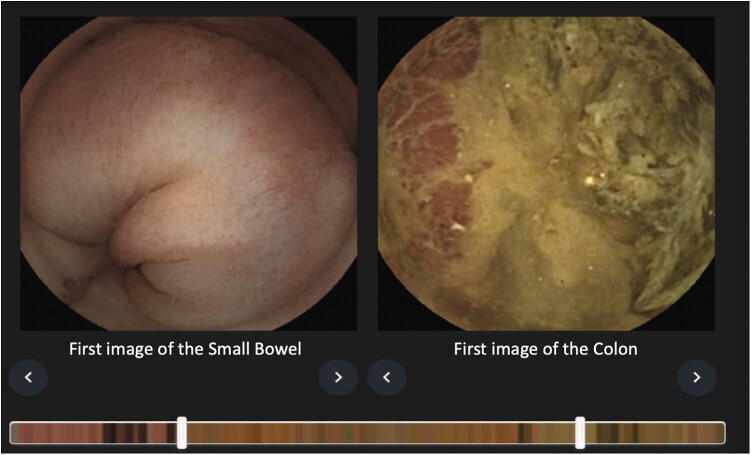
Manual annotations of transition zones on the secure website.

SBCE videos were reviewed on a dedicated online secure website developed by the Nantes Digital Science Laboratory (LS2N). Transition zones were manually annotated by three expert readers (AB, CLB, and RR), each reviewing one-third of the SBCE examinations ( Fig. 1 ). A CSV file was then exported from the platform, in which each image was labeled as “Stomach,” “Small Bowel,” or “Colon”, considered as the ground truth anatomic classification. To assess interobserver reliability, each expert subsequently annotated all examinations they had not originally labeled, enabling formal pairwise agreement analysis for both landmarks.

### Frame-level Classification Task

The primary task of our model was the classification of individual images into the three anatomic categories. To this end, the previously selected ResNet-18 architecture was trained and evaluated using the PyTorch framework. The annotated frame dataset was randomly partitioned into three subsets following a standard 50/25/25 split. The model was initially pretrained on ImageNet and subsequently fine-tuned for anatomic classification. Of note, the SBCE images used in this study did not contain embedded timestamps, and acquisition time was therefore not provided as an input to the model. Each frame was processed independently, without any temporal data, ensuring that the algorithm relied solely on visual image content.

### Viterbi Algorithm

A postprocessing step based on the Viterbi algorithm was applied to segment entire SBCE videos. This dynamic programming approach, derived from the maximum likelihood principle, 21 considers both the class probabilities, taking into account the probability of previous states, and the likelihood of valid transitions between anatomic regions, learned from the training data. In addition, anatomic constraints were imposed by assigning zero probability to retrograde or physiologically implausible transitions (e.g., colon to stomach), thereby ensuring temporally consistent and anatomically realistic predictions.

As transition probabilities for the Viterbi decoding were estimated from the training data, the postprocessing step was embedded within the model evaluation pipeline. Therefore, we used a 5-fold cross-validation framework for training and testing our model segmentation in order to enhance the robustness and reliability of the results. This method consists in partitioning the set of SBCEs into 5 folds, using one-fold as the test set while the remaining folds serve as the training and validation set, and iteratively rotating this arrangement across all folds. Training was conducted over 20 epochs.

### Statistical Analysis

To evaluate the performance of anatomic landmark localization, we calculated the median (interquartile range [IQR]) number of frames between AI and expert detection in the test set. Timestamps associated with each image were subsequently extracted to provide a more clinically interpretable measure of localization error; these timestamps were used for error quantification purposes only and not as model input. Accuracy was evaluated at thresholds of 5 frames, 20 frames, and 1 minute, as determined to be clinically relevant by expert consensus.

A univariate analysis was subsequently performed to identify factors independently associated with reduced accuracy in localizing SB anatomic landmarks. All statistical analyses were conducted using EasyMedStat. Multicollinearity was assessed using the Belsley–Kuh–Welsch technique, while heteroskedasticity and residual normality were evaluated using the Breusch–Pagan and Shapiro–Wilk tests, respectively. The Newey–West correction was applied to account for heteroskedasticity. A *p* -value < 0.05 was considered statistically significant.

## Results

### Patient Characteristics

A total of 60 SBCE videos were obtained from the CROHN-PREP trial; 30 patients (50.0%) had a PEG-based preparation and 30 patients (50.0%) had a simplified preparation (clear liquid diet with 1.5 L of water). Patient characteristics are presented in Table 1 . SBCE was performed for routine disease monitoring in 32 patients (53.3%), for suspected SB involvement in 15 patients (25.0%), for initial assessment of SB involvement at CD diagnosis in 10 patients (16.7%), and in the postoperative setting in 3 patients (5.0%).

**Table 1:** Patient characteristics at baseline.

	Population ( *N* = 60)
Male gender, *n* (%)	28 (46.7)
Age at baseline (years), mean (SD)	26.8 (9.6)
Active smoking, *n* (%)	10 (16.7)
Disease duration (years), mean (SD)	8.5 (9.2)
** Disease location, *n* (%) **	
L1 (ileal)	32 (53.3)
L2 (colonic)	5 (8.3)
L3 (ileocolonic)	23 (38.3)
Upper GI tract involvement, *n* (%)	4 (6.7)
Perineal location, *n* (%)	10 (16.7)
** Disease behavior, *n* (%) **	
B1 (inflammatory)	37 (61.7)
B2 (stricturing)	17 (28.3)
B3 (penetrating)	6 (10.0)
History of intestinal resection, *n* (%)	22 (36.7)
Presence of a stoma, *n* (%)	1 (1.7)
Harvey Bradshaw index, mean (SD)	2.2 (3.0)
Active disease ^a^ , *n* (%)	29 (48.3)
**PRO-2, mean (SD)**	
Stool frequency	1.0 (1.8)
Abdominal pain	0.6 (0.9)

^a^ Defined with a Harvey Bradshaw index >4 or CRP >5 mg/L or fecal calprotectin >100 μg/g or presence of inflammatory lesions at standard endoscopy or imaging performed in the previous 6 months.

Abbreviations: GI, Gastrointestinal; PRO, Patient-reported outcomes; SD, Standard deviation.

The capsule completion rate was high ( *n* = 57, 95.0%), the mean (SD) SBTT was 222.8 (86.3) min, i.e., 3.7 (1.4) h, and the mean (SD) gastric transit time was 35.4 (36.6) min. SBCE findings regarding disease activity, as assessed by the Lewis score, as well as the quality of SB preparation, as quantified by the KODA score, are reported in Table 2 . More than half of the study population ( *n* = 32, 53.3%) had active SB inflammation, defined by a Lewis score ≥135, reflecting the presence of at least one inflammatory lesion – edema, ulceration, or stenosis – within the entire SB. Active inflammation of the terminal ileum was observed in more than one-third of patients ( *n* = 31, 35.0%), defined by a Lewis score ≥135 in the SB segment located upstream of, and adjacent to, the ileocecal valve or the ileocolonic anastomosis.

**Table 2:** SBCE assessment of disease activity (Lewis score) and small bowel preparation quality (KODA score).

	Population ( *N* = 60)
**Small bowel disease activity**	
Overall Lewis score (LS), mean (SD)	508.8 (1038.0)
LS = 0, *n* (%)	24 (40.0)
LS < 135, *n* (%)	3 (5.0)
LS 135–790, *n* (%)	22 (36.7)
LS > 790, *n* (%)	11 (18.3)
**Lewis score (LS) for the third tertile (T3), mean (SD)**	455.7 (1048.5)
T3-LS = 0, *n* (%)	31 (51.6)
T3-LS < 135, *n* (%)	4 (6.7)
T3-LS 135–790, *n* (%)	16 (26.7)
T3-LS > 790, *n* (%)	9 (15.0)
**Small bowel preparation quality**	
Overall KODA score, mean (SD)	2.0 (0.5)
Overall KODA score > 2.25 ^a^ , *n* (%)	21 (35.0)
KODA score for the third tertile (T3), mean (SD)	1.5 (0.8)
T3-KODA score > 2.25 ^a^ , *n* (%)	12 (20.0)

^a^ : Using a previously validated threshold of 2.25, the KODA score accurately discriminates between images with good cleanliness and those with fair or poor cleanliness.

Abbreviations: LS, Lewis score; SBCE, Small bowel capsule endoscopy; SBTT, Small bowel transit time; SD, Standard deviation; T3, Third tertile.

### Training and Test Dataset Size

The model was trained and evaluated on the overall dataset, which comprised 596,980 images, including 426,674 SB images distributed across 5 folds, based on expert annotations. SBCE examinations were randomly assigned to each fold. For each iteration, one fold was used as the test set, while the remaining folds collectively constituted the training set. The distribution of images across the folds is detailed in **Supplementary Table 2** .

### Accuracy of Anatomic Landmark Localization

Interobserver agreement for landmark localization was excellent across all annotator pairs for both the pylorus and the ileocolonic junction ( **Supplementary Table 3** ), supporting the validity of the ground truth annotations used for model training and evaluation.

For each landmark, we computed the number of frames separating AI detection from expert annotation. The results of the 5-fold cross-validation are summarized in **Supplementary Table 4** for both the pylorus and the ileocolonic junction. Across all videos, the model localized the pylorus with a median difference [IQR] of 2.0 [1.0–110.0] frames relative to expert labels, corresponding to a median of 1 [0–86] s. The ileocolonic junction was identified with a median difference of 118.5 [1.0–843.25] frames compared with the expert annotations, corresponding to a median of 312 [1–4547.25] s (i.e., 5 min and 12 s).

When applied to individual images, our model achieved a mean (standard deviation [SD]) accuracy of 0.93 (0.09) across the three anatomic classes for the entire cohort.

### Temporal Discrepancies between AI-based and Expert Identification of Anatomic Landmarks

To assess the potential clinical impact of errors in AI-based anatomic landmark detection, we analyzed the temporal discrepancies between the CNN predictions and expert annotations, distinguishing detections occurring *before* or *after* manual reference annotation.

The pylorus was perfectly identified by the AI model in 9 SBCE videos (15.0%). In 37 videos (61.6%), the CNN detected the pyloric location *before* expert annotation, with a median [IQR] discrepancy of 2.0 [1.0–385.0] frames, corresponding to 2.0 [1.0–267.0] s. Conversely, in 14 SBCE videos (23.3%), pyloric detection by the AI tool occurred *after* manual annotation, with a median delay of 8.5 [3.5–88.25] frames, corresponding to 3.0 [1.0–74.75] s.

Regarding the ileocolonic junction, perfect agreement between the AI model and expert annotation was observed in 13 SBCE videos (21.6%). In 32 SBCE videos (53.3%), the CNN identified the ileocolonic junction *before* expert annotation, with a median [IQR] discrepancy of 544.5 [144.2–2395.0] frames, corresponding to 2780 [318.5–5592.5] s (i.e., 46 min and 20 s), a scenario that could result in missed SB lesions during automated analysis. In 15 videos (25.0%), ileocolonic junction detection occurred *after* manual annotation, with a median frame discrepancy of 12.0 [2.5–592.5], corresponding to 188 [32–2720] s (i.e., 3 min and 8 s).

### Combined Pylorus and Ileocolonic Junction Localization Performance

To evaluate the overall clinical reliability of the AI model, we assessed its performance in localizing both the pylorus and the ileocolonic junction within the same SBCE examinations, considering frame-level discrepancies relative to expert annotations and defining clinically acceptable localization as a temporal difference of less than one minute.

The AI model achieved exact frame-level identification of both anatomic landmarks in 2 SBCEs (3.3%). In 13 SBCEs (21.7%), landmarks were located within five frames, and in 22 SBCEs (36.7%), within 20 frames of the expert reference. When considering a temporal discrepancy of less than one minute as clinically acceptable, the model successfully localized both landmarks in 26 SBCE videos (43.3%).

### Factors Associated with Accurate Localization of the Ileocolonic Junction

Given the model’s suboptimal performance in localizing the ileocolonic junction, we performed a univariate analysis to explore factors potentially influencing accurate localization. For this analysis, we defined “accurate localization” as a frame discrepancy ≤118.5 frames, based on the observed differences from expert annotations reported previously and corresponding to approximately one minute in a mean duration video of 222.8 min, assuming an acquisition rate of two frames per second – the same threshold previously considered clinically acceptable for overall landmark localization.

In univariate analysis, none of the following factors showed a significant association with accurate localization: presence of distal ileitis (odds ratio [OR] = 0.66; 95% confidence interval [CI] 0.24–1.86; *p* = 0.600), prior ileocolonic resection (OR = 0.75; 95% CI 0.26–2.15; *p* = 0.789), incomplete examination (OR = 0.48; CI 0.041–5.63; *p* > 0.999), PEG-based preparation (OR = 1.31; CI 0.47–3.6; *p* = 0.796), adequate bowel cleanliness defined by an overall KODA score >2.25 (OR = 2.88; CI 0.95–8.72; *p* = 0.104), and adequate bowel cleanliness in the third tertile defined by a segmental KODA score >2.25 (OR = 3.27; 95% CI, 0.77–13.83; *p* = 0.181). Multivariate analysis was not performed due to the lack of significant findings in the univariate analysis.

### Expert Review of SBCE Examinations with Poor AI Localization

Recognizing the limitations of the AI model in certain cases, two SBCE readers (RR and AB) jointly reviewed examinations with poor landmark localization to identify contributing factors and better understand the model’s performance. “Poor localization” was defined according to expert-established thresholds, reflecting clinically relevant discrepancies: >1000 frames between AI and expert detection for the ileocolonic junction, and >350 frames for pyloric localization.

The corresponding results are presented in **Supplementary Table 5** . The most common expert interpretations for cases in which the AI model struggled with accurate landmark localization were inadequate bowel cleanliness, capsule stagnation at the ileocolonic junction, and pyloric repetitive back-and-forth movements.

## Discussion

In this study, we evaluated an AI-based model for automated detection of the pylorus and ileocolonic junction in SBCE videos from patients with CD. Our findings demonstrate that a ResNet-18 architecture coupled with a Viterbi algorithm can reliably identify the pylorus, with a median localization error of only 1 s. Performance at the ileocolonic junction was more modest (median localization error of ~5 min), with a substantial proportion of early detections by a median of ~46 min carrying a non-negligible risk of missed terminal ileal lesions. Capsule stagnation, pylorus back-and-forth movements, and suboptimal bowel cleanliness emerged as the main contributors to localization errors.

Several approaches have previously been proposed to automate the identification of anatomic transition zones in SBCE across several indications. 22 23 24 Early methods relied on color-based and texture classifiers designed to detect mucosal transitions, 25 26 with performance later enhanced through image preprocessing steps. 27 The advent of deep CNN brought significant improvements. Zou et al. were among the first to demonstrate the feasibility of frame-level organ classification, achieving 95.5% accuracy across stomach, SB, and colon classes, 28 although without temporal continuity. Son et al. subsequently introduced a ResNet-50-based classifier combined with temporal smoothing, achieving mean localization errors of 38.8 s for the pylorus and 32.0 s for the ileocecal valve. 29 These remarkably low errors should, however, be interpreted with caution: cases in which the capsule did not reach the colon or in which organs could not be visually distinguished were excluded from the analysis; the training pipeline treated normal and abnormal capsule studies differently, which does not reflect real-world clinical conditions; and no data on clinical indication were provided, leaving open the question of whether patients with CD, who may present particular challenges for ileocolonic junction detection, were included. More recently, Nam et al. proposed a sequence-aware hybrid model integrating EfficientNet-B0 with long short-term memory (LSTM) networks, a recurrent architecture that processes frames sequentially through a hidden state, achieving an overall accuracy of 97.1% with mean transition errors of 4.3 and 24.7 min for the gastric and ileocecal landmarks, respectively. 30 Among commercially available AI-enabled SBCE systems, the only published study assessing automated landmark detection reported poor performance, with mean discrepancies of 49.2 min for the first duodenal frame and 99.2 min for the first cecal frame. 31 Our approach combines a ResNet-18-based CNN with a physiologically constrained Viterbi postprocessing algorithm. From a data fusion standpoint, frame-level classification constitutes a single-image baseline, while the Viterbi algorithm operates as a late fusion mechanism enforcing temporal consistency at the sequence level. This is conceptually distinct from early fusion approaches, in which multiple consecutive frames are concatenated as model input, and from recurrent architectures such as LSTMs. We deliberately adopted a simple and interpretable strategy, explicitly integrating temporal continuity in a nonparametric and computationally efficient manner, rather than relying on such sequential modeling techniques or more complex temporal architectures, which would require a dedicated study. Exploring early fusion by feeding short consecutive sequences of frames directly into the model is a promising direction that the literature consistently shows to yield strong performance gains, and is currently being investigated by our group as a natural extension of this work.

Time-based values were derived post-hoc from capsule frame timestamps and are provided solely to aid clinical interpretation; timestamps were deliberately excluded from model inputs to preserve image quality and to demonstrate that the model can learn directly from visual information alone, without relying on temporal cues. Beyond this methodologic choice, absolute time is unlikely to be a reliable predictor of anatomic location, as capsule transit speed varies considerably between patients, particularly in CD, where gastric emptying may be delayed, SB transit altered, and capsule stagnation around anastomotic sites more likely. Furthermore, the transitions targeted in this study correspond to visually defined anatomic landmarks rather than temporally predictable events, and misclassifications predominantly occurred between adjacent classes, suggesting that temporal cues would not have resolved the main sources of error. Whether incorporating timestamps as an additional input could nonetheless improve performance remains an avenue for future investigation.

The pyloric landmark was defined as the first bulbar image, rather than the pyloric orifice itself, which is not consistently visualized as a distinct structure during SBCE. This definition was preferred over the major duodenal papilla, which is visualized inconsistently, if not rarely, precluding its systematic use for automated detection. It also minimizes annotation burden by relying on the identification of a single transitional frame rather than a specific anatomic structure, while remaining clinically meaningful, particularly in patients with CD in whom bulbar lesions may occur.

To our knowledge, this is the first study to report prediction direction as a distinct outcome measure. Prior works have consistently focused on overall localization error without distinguishing whether the model predicted *before* or *after* the expert annotation, an oversight that is clinically consequential, as the direction of error directly determines if lesions risk being missed. Here, prediction direction analysis showed that pyloric detection preceded expert annotation in most cases, with a clinically acceptable median discrepancy of ~3 s when it occurred afterward. For the ileocolonic junction, early detection occurred in 32 cases with a median discrepancy of ~46 min, highlighting a non-negligible risk of missed terminal ileal lesions, and supporting the current use of this model as an assistive rather than fully autonomous tool.

Systematic review of poorly performing examinations revealed that pyloric mislocalization was most frequently associated with inflammatory gastric mucosa mimicking SB mucosa, or with repeated capsule back-and-forth movements around the pylorus, a structural constraint of the Viterbi transition matrix, in which retrograde transitions are assigned zero probability. This reflects an inherent methodological trade-off, as no single strategy is optimal across all possible scenarios. Addressing this issue would require a complete redesign of the model and will be the subject of a dedicated future study. For the ileocolonic junction, capsule stagnation and inadequate distal bowel cleanliness were the main limiting factors. Neither distal ileitis, bowel preparation type, nor KODA score emerged as significant independent predictors of model performance. The inclusion of two preparation regimens from the CROHN-PREP trial should be regarded as a strength, as it exposed the algorithm to a broader range of visual conditions.

Performance at the ileocolonic junction may vary according to clinical indication, as inflammatory lesions or surgical anastomoses could plausibly alter the visual appearance of this region. However, in our cohort, where ileocolonic anastomosis accounted for 36.7% of patients, univariate analysis did not identify any significant association between these factors and detection accuracy, suggesting that the model may be more robust to such anatomic variability than initially anticipated. These findings nonetheless require confirmation in larger, prospective cohorts before conclusions can be drawn regarding indication-specific performance. We fully acknowledge that the proposed model does not yet meet the standards required for autonomous clinical implementation. Reporting these results transparently is essential: negative and mitigated findings delineate the boundaries of current methods and help orient future research toward the directions where progress is most needed.

The present study focused specifically on patients with CD, a deliberate methodological choice, as this population represents particularly challenging conditions for AI-based landmark detection. By validating our algorithm in this worst-case setting, we hypothesize that performance is likely to be replicated or surpassed in less challenging indications. Notably, 40% of patients had a Lewis score of 0, and 25% underwent SBCE for suspected rather than confirmed SB involvement, subgroups closely mirroring non-CD clinical profiles.

Finally, the relatively limited number of video examinations ( *n* = 60) is an inherent constraint of this ancillary trial study; however, our sample size is consistent with already published deep learning studies applied to SBCE, where cohorts have typically ranged around 50–100 videos. 5 6 7 8 9 Our dataset encompassed 596,980 digestive frames, including 426,674 SB images, a substantial volume for frame-level CNN training, which exceeds those reported in prior works. 5 6 7 Furthermore, by combining a 50/25/25 training/validation/test split with five-fold cross-validation, all 60 videos were ultimately included in the test set. Moreover, the targeted task consisted here of identifying SB transition landmarks rather than multiple lesion types, which may require less dataset heterogeneity than broad lesion-detection models.

In conclusion, this study demonstrates that a ResNet-18-Viterbi framework can automate anatomic segmentation in SBCE for patients with CD, with particularly strong performance for pyloric localization. Detection of the ileocolonic junction remains more challenging, especially in case of capsule stagnation and suboptimal distal bowel cleanliness. Nevertheless, the model achieves clinically meaningful accuracy, supporting its potential role as an assistive tool in routine practice, while highlighting the need for further refinement and validation in larger, more diverse cohorts before fully autonomous deployment.

**Abbreviations**
AI: Artificial intelligence
CD: Crohn’s disease
CI: Confidence interval
CNN: Convolutional neural network
IQR: Interquartile range
LSTM: Long short-term memory
OR: Odds ratio
PEG: Polyethylene glycol
SB: Small bowel
SBCE: Small bowel capsule endoscopy
SD: Standard deviation

## Data Availability

The datasets generated and/or analyzed during the current study are available from the corresponding author on reasonable request.

## Supplementary Material

Ergänzendes Material Supplementary Material
