# Supplementary material for: Artificial Intelligence-Based Localization of Small Bowel Anatomic Transition Zones in Crohn’s Disease Using Capsule Endoscopy
Source: Endosc Int Open. 2026 Jun 12;14:a28841351. doi: 10.1055/a-2884-1351 (PMC13289779; doi:10.1055/a-2884-1351)
Supplement: Supplementary file 1 — Ergänzendes Material [file 10-1055-a-2884-1351_28859809.pdf]

Supplementary data:

Artificial Intelligence-Based Localization of Small Bowel Anatomical Transition Zones in Crohn's Disease Using Capsule Endoscopy

Raphaëlle Rouveyre, Tristan Gomez, Harold Mouchère, Arnaud Bourreille, Catherine Le Berre

Supplementary Table 1. Comparison of the AI-based ResNet-18 models tested in the preliminary analysis.

	Accuracy			
	**Global**	**"Stomach"**	**"Small Bowel"**	**"Colon"**
**ResNet-18 model**	90.2%	66.0%	96.0%	84.0%
**ResNet-18 model + optimization Adam**	89.0%	65.0%	96.0%	77.0%
**ResNet-18 model + optimization Adam + MSE Loss**	92.0%	82.0%	97.0%	72.0%
**ResNet-18 model + optimization Adam + MSE Loss + Drop out 0.1 + Weight Decay 10^{-6}**	92.3%	76.0%	97.0%	84.0%

Abbreviations: MSE, Mean squared error.

Supplementary Table 2. **Image dataset distribution across folds according to the test set fold**

	Fold 1 (15 SBCE)	Fold 2 (13 SBCE)	Fold 3 (12 SBCE)	Fold 4 (11 SBCE)	Fold 5 (9 SBCE)
**Training set**					
• **Total number of frames**	445287	466294	478679	507814	490146
• **Number of SB frames**	319919	333379	334794	368326	350278
**Test set**					
• **Total number of frames**	151693	130686	118301	89166	106834
• **Number of SB frames**	106755	93295	91880	58348	76396
**Overall dataset**					
• **Total number of frames**			596980		
• **Number of SB frames**			426674		

Abbreviations: SB, Small bowel; SBCE, Small bowel capsule endoscopy.

Supplementary Table 3. Interobserver agreement for anatomical landmark localization

Landmark	Annotator pair	Spearman ρ	R^2	ICC	95% CI	p
Pylorus	AB – CLB	0.94	0.916	0.95	[0.931 – 0.970]	<0.001
	AB – RR	0.94	0.922	0.96	[0.938 – 0.973]	<0.001
	CLB – RR	0.98	0.989	0.99	[0.982 – 0.992]	<0.001
Ileocolonic junction	AB – CLB	0.98	0.928	0.96	[0.945 – 0.976]	<0.001
	AB – RR	0.95	0.862	0.93	[0.892 – 0.953]	<0.001
	CLB – RR	0.96	0.922	0.96	[0.936 – 0.973]	<0.001

Abbreviations: ICC, Intraclass correlation coefficient; CI, Confidence interval

Supplementary Table 4. Anatomical landmark localization results from the 5-fold cross-validation of the model

	Pylorus localization, median [IQR]	Ileocolonic junction localization, median [IQR]
**Fold 1 (15 SBCE)**	3.0 [2.0–9.5]	343.0 [4.5–514]
**Fold 2 (13 SBCE)**	2.0 [1.0–92.0]	76.0 [0.0–2309.0]
**Fold 3 (12 SBCE)**	1.0 [0.7–54.7]	131.5 [1.5–1200.7]
**Fold 4 (11 SBCE)**	2.0 [1.0–8.5]	6.0 [0.5–687.5]
**Fold 5 (9 SBCE)**	104.0 [2.0–414.0]	753.0 [2.0–964.0]

Abbreviations: IQR, Interquartile range; SBCE, Small bowel capsule endoscopy.

Supplementary Table 5. Individual assessment of SBCE examinations with poor AI model performance

SBCE ID (pseudo-nymized)	Fold	Pylorus (frame number)		Ileocolonic junction (frame number)		Expert interpretation
		AI-based prediction	Expert manual annotation	AI-based prediction	Expert manual annotation	
**Poor AI-based localization for both pylorus and ileocolonic junction**						
01-066	3	416	801	9852	15232	Inadequate cleanliness (T3); pylorus repetitive back-and-forth movements; capsule stagnation at the ileocecal valve
01-026	3	70	1113	9882	7299	Endoscopic appearance of gastritis
01-016	4	922	1284	10282	9044	Endoscopic appearance of gastritis; capsule stagnation at the ileocecal valve
01-036	5	63	7456	12702	15774	Endoscopic appearance of gastritis, pylorus repetitive back-and-forth movements
01-059	5	1122	2564	7743	10153	Pylorus repetitive back-and-forth movements
**Poor AI-based localization for ileocolonic junction**						
01-021	1	495	499	7783	13672	Inadequate cleanliness (T3); presence of blood; incomplete SBCE examination
01-027	1	1190	1185	6760	8022	Inadequate cleanliness (T3)
01-054	2	279	281	7634	10688	Capsule stagnation at the ileocecal valve
01-001	2	1952	1951	4165	6474	No identifiable explanatory factor
01-045	2	978	979	2073	4463	Inadequate cleanliness (T3)
01-047	3	842	842	12344	14882	Inadequate cleanliness (T3)
01-044	4	1036	1033	8155	11995	Abundant presence of bile (T3)
01-023	4	135	137	5603	4101	Capsule stagnation at the ileocecal valve

Abbreviations: AI, Artificial intelligence; SBCE, Small bowel capsule endoscopy; T3, Third tertile.
